# Type of bacterial isolates and antimicrobial resistance profile from different clinical samples at a Referral Hospital, Northwest Ethiopia: five years data analysis

**DOI:** 10.1186/s13104-019-4604-6

**Published:** 2019-09-11

**Authors:** Melkamu Abebe, Senait Tadesse, Girum Meseret, Awoke Derbie

**Affiliations:** 1 Amhara Public Health Institute (APHI), Bahir Dar, Ethiopia; 2 Department of Medical Laboratory Science, College of Health Science, Bahir Dar University, Bahir Dar, Ethiopia; 3 The Centre for Innovative Drug Development and Therapeutic Trials for Africa (CDT-Africa), Addis Ababa University, Addis Ababa, Ethiopia

**Keywords:** Bacterial profile, Antimicrobial resistance, Debre Markos, Ethiopia

## Abstract

**Objective:**

Antimicrobial resistance (AMR) is one of the most serious global public health threats that exert a significant burden in terms of patient morbidity and mortality and financial crises in many developing countries including Ethiopia. Knowledge on the type of predominantly circulating pathogens with their respective AMR profile in a given area is essential for optimal patient care. This study was aimed at assessing the types of bacterial isolates and their AMR profile identified from a range of clinical samples at Debre Markos Referral Hospital, Northwest Ethiopia, over a period of 5 years (2013 to 2017).

**Results:**

From the total of 514 different clinical samples processed in the stated time frame, about 240 (46.7%) yield bacterial growth. Majority of the identified bacteria were isolated from stool culture 68 (28.3%) followed by urine 56 (23.3%), ear discharge 54 (22.5%) and wound swabs at 26 (10.8%). Most of the clinical isolates were Gram-negative at 171 (71.25%). The predominant isolate was *S. aureus* at 41 (17.1%) followed by *Salmonella* species, 40 (16.7%), *Escherichia coli* 36 (15%) and *Pseudomonas aeruginosa* at 26 (11.7%). Generally, the isolates were found resistant at (60–100%) against ampicillin, co-trimoxazole, doxycycline, gentamicin, norfloxacin and tetracycline. Gram-positive isolates were found relatively sensitive to ceftriaxone, erythromycin and vancomycin at (71–84%).

## Introduction

Antimicrobial resistance (AMR) is a phenomenon in which microorganisms become resistant to antimicrobial agents to which they were originally sensitive [1]. The major mechanism of antimicrobial resistance is the result of a specific evolutionary pressure to develop a counter attack mechanism against an antimicrobial or class of antimicrobials [2]. Antimicrobial resistance places a significant burden on everyone, both in terms of patient morbidity and financial cost. A high percentage of hospital acquired infections and medical complications are widely common around the globe due to an increasing AMR pathogens, yet the issue received little concern by health care sectors [3].

In developing countries like Ethiopia, where there are huge infrastructural and regulatory challenges, antimicrobial resistance is widely available and the problem is deep rooted [4]. An elevated AMR among gastrointestinal pathogens as well as increased and multiple resistance rates to erythromycin (89.4%), amoxicillin (86.0%) and tetracycline (72.6%) have been documented among isolates from urine, ear discharge, pus swab from wounds, and eye discharge. Isolates from the cerebrospinal fluids (CSF) as well as urinary pathogens have demonstrated multidrug resistance as well [5].

The problem of the antimicrobial resistance is not only the development of the resistance but also the transmission of the resistant strains from one person to another, especially in the health facility setting. The transmission may occur due to the day to day interrelations of people or the movement of animals or with different types of packed foods and drinks. The problem gets worsen in countries like Ethiopia where poor sanitation make easy for the bacteria to transmit.

In Ethiopia the treatment of most of bacterial infections is made usually empirically in which the etiologic agents are rarely identified. So, identifying the most common bacterial pathogens and their respective AMR profile would be valuable to optimize treatment and ultimately to reduce morbidity and mortality associated with infectious disease. Therefore, this study was conducted aimed at assessing the type of pathogenic bacterial isolates and their antimicrobial resistance profile from different kinds of clinical samples at Debre Markos Referral Hospital, northwest Ethiopia.

## Main text

### Methods

The study was conducted at Debre Markos referral hospital (DMRH) which is located in Northwest part of Ethiopia at 300 km away from the nation’s capital, Addis Ababa. The hospital has been serving for about 3.5 million people of Debre Markos and its surroundings for the last 50 year. Even though, bacteriological culture and antimicrobial susceptibility testing (AST) was started since 2011 in the hospital, well recorded bacteriological data was noted since 2013.

A retrospective cross-sectional study was conducted at DMRH medical microbiology laboratory. Bacteriological data recorded in the period of November 2013 to February 2017 were retrieved for analysis using prepared data extraction sheet. Patient related data (age and sex) with full record of bacteriological culture and antimicrobial resistance profile were retrieved from the laboratory registration book. A total of 514 specimens were collected from 514 patients during the stated time period and sent to the DMRH medical microbiology laboratory for bacteriological culture and AST. All kinds of collected clinical specimens were processed based on the recommended microbiology procedures. The Kary-Bauer disc diffusion method was used for AST on Muller Hinton agar (MHA) (Oxoid, Ltd, England) as per the Clinical Laboratory Standards Institute (CLSI) guideline [6]. The performances of culture media were tested by using standard reference strain; *Staphylococcus aureus* (ATCC25923), *Escherichia coli* (ATCC25922) and *P. aeruginosa* (ATCC 27853). After data were checked for completeness and consistencies, they were entered and analyzed using SPSS version 23. Descriptive statistics were used to describe the demographic characteristics of the participants and the bacteriological and antimicrobial resistance profile of the isolates.

### Results

#### The frequency and type of bacterial isolates

Of the total 514 participants, females constituted at 51.8%. Most of the patients were in the age group of 16–45 years at 282 (54.9%), Table 1.

**Table 1:** Age and sex distribution of the study participants at DMRH, 2013–2017

	Sex		Total
	Male	Female	
Age group			
< 15	118 (60.2%)	78 (39.8%)	196 (38.1%)
16–45	106 (37.6%)	176 (62.4%)	282 (54.9%)
> 46	24 (66.7%)	12 (33.3%)	36 (7.0%)
Total	248 (48.2)	266 (51.8%)	514 (100%)

Among the different clinical samples sent to the laboratory during the time frame, a total of 240 (46.7%) were positive for bacterial growth. Majority of the bacteria were isolated from stool culture at 68 (28.3%) followed by urine 56 (23.3%), ear discharge 54 (22.5%) and wound swabs 26 (10.8%). In this study, most of the identified isolates were Gram-negative at 171 (71.25%) while the remaining at 69 (28.75%) were Gram-positives. The overall distribution of the isolates from each kind of clinical samples is summarized in Table 2. The most frequently identified isolate was *S. aureus* at 41 (17.1%) followed by *Salmonella* species 40 (16.7%), *E. coli*, 36 (15%) and *P.* aeruginosa at 26 (11.7%).

**Table 2:** The distribution of identified bacterial pathogenes from different clinical samples at DMRH, 2013–2017

Type of the isolates	Types of the specimens								
	Urine	Stool	Blood	Ear. Dis	Wound	V.dis	U.dis	CSF	Total N (%)
*E. coli*	30 (56.6%)	0	6 (50%)	0	0	0	0	0	36 (15%)
*K. pneumoniae*	16 (30.2%)	0	6 (50%)	0	0	0	0	0	22 (9.1%)
*Shigella sp*	0	28 (41.2%)	0	0	0	0	0	0	28 (11.7%)
*Salmonella sp*	0	40 (58.8%)	0	0	0	0	0	0	40 (16.7%)
*Enterobacter sp*	3 (13.0%)	0	0	0	0	0	0	0	3 (1.25%)
*P. aeruginosa*	4 (7.5%)	0	0	18 (100%)	6 (100%)	0	0	0	28 (11.7%)
*N. meningitidis*	0	0	0	0	0	0	0	2 (100%)	2 (0.83%)
*N. gonorrhoeae*	0	0	0	0	0	8 (100%)	4 (100%)	0	12 (5%)
G-ve bacteria total	53 (94.6%)	68 (100%)	12 (60%)	18 (33.3%)	6 (23.1%)	8 (100%)	4 (100%)	2 (50%)	171 (71.25%)
*S. aureus*	3 (100%)	0	4 (50%)	22 (61.1%)	12 (41.2%)	0	0		41 (17.1)
*S. pneumoniae*	0	0	2 (25%)	6 (16.7%)	0	0	0	2 (100%)	10 (4.2%)
*S. pyogenes*	0	0	2 (25%)	8 (22.2%)	8 (30.8%)	0	0	0	18 (7.5%)
G+ ve bacteria total	3 (5.4%)	0	8 (40%)	36 (66.7%)	20 (66.9%)	0	0	2 (50%)	69 (28.57%)
Total	56 (23.3%)	68 (28.3%)	20 (8.3%)	54 (22.5%)	26 (10.8%)	8 (3.3%)	4 (1.7%)	4 (1.7%)	240 (100%)

*dis* discharge, *CSF* cerebrospinal fluid

From urine culture, Gram-negative isolates at 53 (94.6%) were predominant. Of these, *E. coli* at 30 (56.6%) followed b*y K. pneumoniae* at 16 (30.2%) were the common ones. Similarly, S*almonella* and *Shigella* species were identified from stool cultures.

Gram positive bacteria were the major cause of ear infection in the study area that accounts at 36 (66.7%). Among these, *S. aureus* at 22 (61%) was the major isolate followed by *S. pyogenes* 18 (33.3%), *P. aeruginosa* and *S. pneumoniae* which accounted at 8 (22.2%) and 6 (16.7%) shares, respectively. Further, out of the 26 bacteria isolated from wound/pus swabs, majority of them were Gram-positives at 20 (66.9%).

#### Antimicrobial resistance profile of the isolates

The overall AMR profile of the isolates is presented in Table 3. In this study, the resistance rate of Gram-negatives for amoxicillin, amoxicillin clavulanic acid, ceftriaxone, chloramphenicol, ciprofloxacin, doxycycline, gentamicin, norfloxacin, tetracycline and trimethoprim/sulfamethoxazole was between 41% and 74%. On the other hand, Gram-positive isolates were found sensitive to vancomycin at 84%, erythromycin at 75.3% and ceftriaxone at 71%. We noted that all type of the isolates were found 100% resistant to ampicillin.

**Table 3:** Antimicrobial resistance profile of the isolated organisms at DMRH, 2013–2017

Type of the isolates		Tested antimicrobial agents										
		NOR n (%)	TET n (%)	AMP n (%)	CIP n (%)	SXT n (%)	CRO n (%)	CHF n (%)	DOX n (%)	CN n (%)	AMC n (%)	AMO n (%)
G −ve isolates												
*E. coli* (36)	R	20 (56)	24 (67)	36 (100)	19 (53)	22 (61)	13 (36)	16 (44)	17 (47)	15 (42)	15 (42)	15 (42)
*Klebsiella* sp. (22)	R	11 (50)	18 (82)	22 (100)	9 (41)	17 (77)	8 (36)	10 (46)	12 (55)	10 (46)	9 (40.9)	13 (59)
*Shigella* sp. (28)	R	20 (71)	21 (75)	28 (100)	12 (43)	24 (86)	13 (46)	22 (79)	20 (71)	24 (86)	13 (46)	15 (54)
*P. aeruginosa* (28)	R	21 (75)	24 (86)	28 (100)	14 (50)	24 (86)	15 (54)	17 (61)	22 (79)	12 (43)	11 (39)	18 (64)
*Salmonella* sp. (40)	R	25 (63)	30 (75)	40 (100)	18 (45)	20 (50)	16 (40)	22 (55)	24 (60)	19 (48)	21 (53)	22 (55)
*Enterobacter* sp. (3)	R	1 (33)	2 (67)	3 (100)	1 (33)	1 (33)	3 (100)	1 (33)	1 (33)	1 (33)	1 (33)	1 (33)
*N. gonorrhoeae* (12)	R	5 (42)	7 (58)	–	6 (50)	7 (58)	2 (17)	9 (75)	8 (67)	9 (75)	9 (75)	9 (75)
*N. meningitidis* (2)	R	1 (50)	1 (50)	–	1 (50)	1 (50)	0	1 (50)	1 (50)	1 (50)	1 (50)	1 (50)
Total (171)	R	104 (60)	127 (74)	157 (100)	80 (47)	116 (68)	70 (41)	98 (57)	105 (61)	110 (64)	80 (47)	94 (55)

									CL n (%)	VAN n (%)	P n (%)	ERY n (%)
G+ isolates												
*S. aureus* (41)	R	30 (73)	26 (63)	41 (100)	20 (49)	18 (44)	15 (37)	23 (56)	22 (54)	5 (12)	15 (37)	17 (42)
*S. pyogenes* (18)	R	15 (83)	14 (78)	18 (100)	13 (72)	14 (78)	10 (56)	12 (67)	11 (61)	4 (22)	6 (33)	8 (44)
*S. pneumoniae* (10)	R	6 (60)	7 (70)	10 (100)	5 (50)	6 (60)	4 (40)	4 (40)	6 (60)	2 (20)	3 (30)	2 (20)
Total (69)	R	41 (59)	47 (68)	69 (100)	38 (55)	38 (55)	29 (42)	39 (57)	39 (57)	11 (16)	24 (35)	27 (39)

*R%* resistance rate, *NOR* norfloxacin, *TET* tetracycline, *AMP* ampicillin, *CIP* ciprofloxacin, *SXT* trimethoprim/sulfamethoxazole, *CRO* ceftriaxone, *CHF* chloramphenicol, *DOX* doxycycline, *CN* gentamicin, *AMC* amoxicillin clavulanic acid, *AMO* amoxicillin, *CL* clindamycin, *VAN* vancomycin, *P* penicillin, *ERY* erythromycin

### Discussion

In most resource limited settings the emergence and spread of multi-drug resistant pathogens is one of the major challenges for the provision of good quality health service in hospitals. Successful management of patients with different kinds of infectious diseases depends on the identification of the bacterial pathogens and on the proper selection of antimicrobials effective against the organisms. This study was conducted to assess the distribution of bacterial pathogens and by extension to evaluate their antimicrobial resistance profile from different kinds of clinical specimens at a referral hospital in Ethiopia over 5 years period.

In the present study, the overall proportion of culture positive result was at 46.7%. Of all isolates, Gram-negative at 71% were more frequent. It is well articulated that Gram-negatives are predominantly isolated in different clinical specimens especially they are an important cause of nosocomial infections (sepsis, pneumonia, and meningitis) because they generally cause severe disease. According to previous reports, the presence of multi-drug resistant strains of these types of isolates has been associated with prolonged hospital stays, higher health care costs and an increased morbidity and mortality in resource limited settings, including Ethiopia [1, 7–13].

In the present study, majority of the clinical isolates were recovered from stool, urine, ear discharge and wound swab cultures. Specifically, among the urine culture isolates, *E.* coli and *K. pneumoniae* were the major identified etiologic agents. This finding is in agreement with other studies done in Ethiopia; at Jimma [7] and Adigrat [8], and Sudan [9]. The gastrointestinal tract gram negative flora claimed to be the well-established causes of most urinary tract infections globally. With regard to the antimicrobial resistance profile of these isolates, they were found fully resistant (100%) to ampicillin. Other similar studies in different part of the country [7, 8, 10] have also reported comparable finding on this regards. In our study, specifically the antimicrobial resistance status of *E. coli* was between 36% and 55% to amoxicillin, ceftriaxone, chloramphenicol, ciprofloxacin, co-trimoxazole, gentamicin, norfloxacin. This report is found to be in line with other reports in Ethiopia and overseas [7, 9, 11, 12]. Similarly, the *K. pneumoniae* which is isolated from urine and blood culture were found resistant at 36%, 45%, 50%, 77%, and 81.8% to ceftriaxone, gentamicin, norfloxacin, co-trimoxazole and tetracycline, respectively. This finding is relatively higher as compared to other reports done in Gondar, Ethiopia [11] and Sudan [9]. Our report support the argument that *K. pneumoniae* is one of most known causes of nosocomial infections and is also well known for its high level antimicrobial resistance including the production of extended-spectrum β-lactamases (ESBLs) by most strains in most health facility settings around the world.

In our study, one of the stool culture isolate was *Shigella* spp. which was found resistant for tetracycline, chloramphenicol, co-trimoxazole, and gentamicin at 75%, 78%, 86% and 86%, respectively. This level of resistance might be related with a high rate of prescription and self-medication of these drugs in the study area. A similar survey conducted in the country at the Gondar university hospital had reported almost same findings with our report [13]. Likewise, in the present study the *P. aeruginosa* which isolated from urine, ear discharge and wound swabs was found resistant to ciprofloxacin, chloramphenicol, tetracycline, co-trimoxazole, ceftriaxone, norfloxacin at 50%, 60%, 86%, 86%, 53% and 75%, respectively. Other studies done in Ethiopia and abroad have also reported comparable finding [9, 14, 15].

The two *N. meningitidis* isolates identified from CSF showed a significant level of resistance to amoxicillin, ciprofloxacin, chloramphenicol and doxycycline. However, both isolates were found sensitive to ceftriaxone. Except, the chloramphenicol resistance figure, our result was found higher than a report in Gondar, Ethiopia [16] which might reflect the continued increasing of antimicrobial resistance in the country.

In our study, we noted that *S. aureus* was isolated from urine, ear discharge, blood and wound swab cultures. This isolate was found resistant between 12% and 100% against ampicillin, ceftriaxone, chloramphenicol, co-trimoxazole, doxycycline, erythromycin, norfloxacin, penicillin, tetracycline and vancomycin. We identified almost similar findings from studies in Ethiopia and overseas [9, 11, 15, 17]. It is known that *S. aureus* is one of the most common bacterial pathogens in most settings and it is a well-known multi-drug resistant pathogen causing different kinds of infections. The overall burden of staphylococcal disease particularly that caused by methicillin resistant *S. aureus* is increasing in many countries in both healthcare and community settings [18].

In Ethiopia the emergence and spread of multi-drug resistant pathogens like *S. aureus,* is shared by the widespread and misuse of antimicrobials by patients due to lack of access to appropriate bacteriological diagnosis and AST services. Under use of drugs due to inadequate dosing or incomplete treatment courses might also contribute its big share for the development of antimicrobial resistance in the country. On top of these, in Ethiopia most of the commonly prescribed antimicrobials are freely available in local pharmacies. Thus, it is quite common to buy and use antimicrobials and other drugs from private pharmacies without prescription [19–23]. These all could play a major role in the increasing trend of antimicrobial resistance in the country in general and the present study area in particular.

### Conclusions

In this study authors provided 5 years bacteriological and AMR data of the DMRH. The major proportion at 71% of the isolates were Gram-negatives which found fully resistant to ampicillin and greater than 60% resistance was noted against co-trimoxazole, doxycycline, gentamicin, norfloxacin, tetracycline. Therefore, as there is no well-standardized bacteriological and AMR surveillance system in the study area, regular monitoring of the etiologic agents and their antibiotic resistance profile should be evaluated for better patient management. Moreover, actions to contain the impact of AMR should be evaluated and strengthened in the study area.

## Limitations of the study

Since the study was relied on secondary data that doesn’t bear most patient related histories, it was not possible to show the detail clinical profile of the study participants. In line with this, we also acknowledge the limitation of such secondary data for some level of discrepancies observed in the resistance profile of ampicillin and amoxicillin, specifically, among stool isolates. Moreover, the study didn’t provide information on the extended-spectrum beta-lactamase status of the isolates.

## Data Availability

The finding of this study is generated from the data collected and analyzed based on the stated methods and materials. All generated data are included in the manuscript. The original data set supporting this finding will be available at any time upon request

## Abbreviations

AMR: antimicrobial resistance
AST: antimicrobial sensitivity testing
ATCC: American Type Culture Collection
CSF: cerebrospinal fluids
DMRH: Debre Markos referral hospital
SPSS: Statistical Package for Social Science

## References

[1] Hart CA, Kariuki S (1998). Antimicrobial resistance in developing countries. BMJ.

[2] Rachakonda S, Cartee L (2004). Challenges in antimicrobial drug discovery and the potential of nucleoside antibiotics. Curr Med Chem.

[3] Allen HK, Donato J, Wang HH, Cloud-Hansen KA, Davies J, Handelsman J (2010). Call of the wild: antibiotic resistance genes in natural environments. Nat Rev Microbiol.

[4] Amabile-Cuevas CF, Jini J (2010). Global perspectives of antibiotic resistance. Antimicrobial resistance in developing countries.

[5] Kimang’a AN (2012). A situational analysis of antimicrobial drug resistance in Africa: are we losing the battle?. Ethiop J Health Sci.

[6] CLSI (2016). Performance standards for antimicrobial susceptibility testing CLSI supplement M100S.

[7] Beyene G, Tsegaye W (2011). Bacterial uropathogens in urinary tract infection and antibiotic susceptibility pattern in Jimma University specialized hospital, southwest ethiopia. Ethiop J Health Sci.

[8] Tadesse S, Kahsay T, Adhanom G, Kahsu G, Legese H, Derbie A (2018). Prevalence, antimicrobial susceptibility profile and predictors of asymptomatic bacteriuria among pregnant women in Adigrat General Hospital, Northern Ethiopia. BMC Res Notes.

[9] Mukhtar AM, Saeed HA (2011). Profile of antibiotic sensitivity and resistance of some pathogenic bacteria isolated from clinical specimens in Sudan. J Sci Technol.

[10] Derbie A, Hailu D, Mekonnen D, Abera B, Yitayew G (2017). Antibiogram profile of uropathogens isolated at Bahir Dar regional health research laboratory centre, northwest Ethiopia. Pan Afr Med J.

[11] Dagnew M, Yismaw G, Gizachew M, Gadisa A, Abebe T, Tadesse T, Alemu A, Mathewos B (2013). Bacterial profile and antimicrobial susceptibility pattern in septicemia suspected patients attending Gondar University Hospital, Northwest Ethiopia. BMC Res Notes.

[12] Kibret M, Abera B (2011). Antimicrobial susceptibility patterns of *E. coli* from clinical sources in northeast Ethiopia. Afr Health Sci.

[13] Yismaw O, Negeri C, Kassu A (2006). A five-year antimicrobial resistance pattern observed in Shigella species isolated from stool samples in Gondar University Hospital, northwest Ethiopia. Ethiop J Health Dev.

[14] Sewunet T, Demissie Y, Mihret A, Abebe T (2013). Bacterial profile and antimicrobial susceptibility pattern of isolates among burn patients at Yekatit 12 hospital burn center, Addis Ababa, Ethiopia. Ethiop J Health Sci.

[15] Tesfaye T, Beyene G, Gelaw Y, Bekele S, Saravanan M (2013). Bacterial profile and antimicrobial susceptibility pattern of external ocular infections in Jimma University specialized hospital, Southwest Ethiopia. Am J Infect Dis Microbiol.

[16] Mulu A, Kassu A, Tassema B (2005). Bacterial isolates from cerebrospinal fluids and their antibiotic susceptibility patterns in Gondar University Teaching Hospital, Northwest Ethiopia. Ethiop J Health Dev.

[17] Wasihun AG, Zemene Y (2015). Bacterial profile and antimicrobial susceptibility patterns of otitis media in Ayder Teaching and Referral Hospital, Mekelle University, Northern Ethiopia. SpringerPlus.

[18] Chambers HF, DeLeo FR (2009). Waves of resistance: *Staphylococcus aureus* in the antibiotic era. Nat Rev Microbiol.

[19] Dinbere T, Bereded F, Derbie A, Biadglegne F (2019). Bacteriology of bacteriology of community acquired pneumonia in adult patients at Felege Hiwot Referral Hospital, Northwest Ethiopia: a cross-sectional study. Antimicrob Resist Infect Control.

[20] Derbie A, Mekonnin D, Abate E (2018). Bacterial isolates and their current drug susceptibility profile from urine among asymptomatic pregnant women attending at a Referral Hospital, Northwest Ethiopia; cross-sectional study. Ethiop J Reprod Health.

[21] Getachew H, Derbie A, Mekonnin D (2018). Surfaces and air bacteriology of selected wards at a referral hospital, Northwest Ethiopia: a cross-sectional study. Int J Microbiol.

[22] Hailu D, Derbie D, Mekonnen D, Zenebe Y, Adem Y, Worku S, Biadglegne F (2016). Drug resistance patterns of bacterial isolates from infected wounds at Bahir Dar Regional Health Research Laboratory Center, Northwest Ethiopia. Ethiop J Health Dev.

[23] Worku S, Gelaw A, Abera Y, Muluneh D, Derbie A, Biadglegne F (2017). Bacterial etiologies, antibiotic susceptibility patterns and risk factors among patients with ear discharge at the University of Gondar Hospital, Northwest Ethiopia. Asian Pac J Trop Dis.

